# Four Cases of Appendicitis, Illustrating Four Types of the Disease*Read at meeting of East Texas Medico-Chirurgical Society; May 31, 1901.

**Published:** 1901-09

**Authors:** A. L. Hathcock

**Affiliations:** Palestine, Texas


For Texas Medical Journal.

### Four Cases of Appendicitis, Illustrating Four Types of the Disease.*

BY A. L. HATHCOCK, M. D., PALESTINE, TEXAS.

So much has been said and written of appendicitis that it is scarcely possible to say anything new upon the subject, but its frequent occurrence, the great uncertainty of its course, its rather frequent innocent appearance, and its only too frequent disastrous result, makes it a subject full of interest to every physician and surgeon, as well as to the layman.

In this paper, which I will make as brief as possible, I wish to present, in the order of their gravity, several cases of appendicitis

*Read at meeting of East Texas Medico-Chirurgical Society; May 31, 1901.

which have occurred in my practice, and which I have selected because they each represent one of the recognized types of the disease and furnish a picture of its phases from the mild to the malignant form.

CASE I. CHRONIC RECURRING APPENDICITIS.

A. H., age 19, male. I saw this case first in August, 1896, at which time he gave a history of attacks of severe colicky pains, attended with vomiting, fever, constipation, and tenderness located in the right illiac fossa. The last of the attacks lasted three weeks and was very severe, the temperature running about 104° F. for two weeks. There was more or less griping and pain in abdomen at all times, and he had constantly to use laxatives in order to secure an action. I advised an operation, but it was deferred, and I did not see him again until October, 1898. In the mean time he had another very severe attack, and several milder ones. The condition was similar to that already stated. No tumor could be felt, but there was great tenderness in the right side.

On October 22, 1898, the appendix was removed, and he had a good recovery, retarded by suppuration of the abdominal wound. due probably to infected catgut.

The appendix was two and one-half inches long, about the size of a lead pencil, with thick, hard walls and a very small cavity. The extreme tip was slightly dilated, and contained a very small amount of mucus. There was no pus.

CASE II. CHRONIC SUPPURATIVE ENDO-APPENDICITIS.

W. G., male, age 22. Consulted me in June, 1901, with this history: About two years ago he was attacked with pain in abdomen and some nausea, lasting about half an hour. During the following eighteen months he had several light attacks, none of them severe enough to put him to bed, and a diagnosis was not made. About six months ago he had the first severe attack, which came on while he was chopping wood. This was attended by severe pain, nausea and vomiting, followed by fever and localization of the pain and tenderness in the right illiac fossa. The muscles were hard on the right side of the abdomen, and the side was also distended somewhat with gas. No tumor could be felt, although an obscure thickening could be made out.

After several attacks like the above, he finally consented to an operation, and the appendix was removed on March 2nd, through an opening made by cutting through the skin and fascia and separating the different muscular layers in the direction of their fibers and afterwards closing with catgut throughout.

The appendix had a few light adhesions, and was easily removed. Its proximal end was completely closed and sclerotic; its distal end was dilated and contained about a teaspoonful of pus.

The recovery was rapid and satisfactory.

### CASE III. ACUTE SUPPURATIVE APPENDICITIS.

A. T., male, age 55. I saw this case in consultation with Drs. F. B. Moore and I. P. Poynor, of this county, in March, 1895. Three weeks prior to my visit he was attacked with the usual symptoms of acute appendicitis, viz., sudden acute pain in the abdomen, vomiting, fever, localized tenderness in right illiac region, and later rigors, sweats, foul tongue, and a large, hard tumor, very tender and painful, in right side, with a temperature of 102°-104° F.

This case was ten miles in the country, and the surroundings rather unfavorable for an operation. It appeared to all of us to be the only thing to do, however, and the abdomen was opened, entering the cavity at the lower border of the tumor, and after passing the fingers around the mass in order to outline it, the incision was enlarged upward and backward, after stuffing gauze into the opening into the abdominal cavity. Deepening the incision, an abscess was opened at the outer side of the colon, the patient turned on the right side, the cavity washed out and explored. It was very large, running upward behind the colon to the liver and downward to the brim of the true pelvis. The appendix was embedded in a large mass of adhesions, and was never seen. After a hurried search for it, we concluded to leave it, as the patient was in rather bad condition. The cavity was packed with iodoform gauze, the wound sutured with silk, except where the drain came out and the patient returned to bed. He had an easy recovery, with a moderate ventral hernia following.

### CASE IV. ACUTE PERFORATING APPENDICITIS.

J. A. R., male, age 22. I was called to see this case late in the afternoon of July 31, 1897. He had a very severe attack of acute appendicitis, six months previously, when an abscess was supposed to have ruptured into the bowel, and he was told by his physician that he would never have any more trouble. I found him in great pain, vomiting, a great deal of tenderness in right side of abdomen, and rigid muscles. The temperature was 101° F., pulse 110. The outlook was stormy from the first. I saw him a few hours later, and all the symptoms were aggravated. The following morning the temperature was 103°, pulse 115, and the tenderness extended up nearly to the ribs. I asked Dr. W. G. Jameson to see this case with me, and it was decided to operate at once. He was taken to the I. & G. N. Hospital, the abdomen prepared and opened about twenty hours from the beginning of the attack. Many dense adhesions were encountered, and the appendix had an opening very close to its base which was leaking pus and fecal matter into the abdomen. The perforation was so close to the coecum that there was not sufficient room for a satisfactory ligation of the stump, which was inverted by Lambert sutures and these reinforced by an additional row, turning the stump still further from the coecum. The tissues were very friable, and I felt much apprehension in regard to the stump. The abdomen was flushed with saline solution, a drain introduced down to the position of the stump, and the wound closed around it. On the second day fecal matter made its appearance around the drain, but otherwise the case progressed favorably. The discharges lost their fecal odor after three weeks, the fistulous tract closed, and the patient's recovery was satisfactory in every respect.

I have arranged for three of these four cases to be present today, in order that the result of the abdominal incisions may be observed. One of the cases, No III, you see has a ventral hernia. This case had a large abscess, and the cavity was kept packed with gauze about two weeks.

Case I shows that infection occurred during the healing process. On the fourth day the temperature ran up to 102° F., with quickened pulse and pain in the wound. A stitch was removed, and a half ounce of pus liberated from the depth of the wound. I attributed this infection to bad gut used in suturing the muscles.

Case II shows a linear wound which was closed throughout with gut. The healing was rapid and complete.

Palestine, Texas, May 31, 1901.

